# Management of Unrepaired Tetralogy of Fallot in an 86-Year-Old Patient Undergoing Transcatheter Aortic Valve Replacement

**DOI:** 10.7759/cureus.52987

**Published:** 2024-01-26

**Authors:** Armish Singh, Samantha Arzillo, Kevin Ergle

**Affiliations:** 1 Anesthesiology, HCA Florida Westside Hospital, Plantation, USA

**Keywords:** transcatheter aortic valve replacement (tavr), eisenmenger syndrome, aortic stenosis (as), tetrology of fallot, adult congenital heart disease (achd)

## Abstract

Tetralogy of Fallot (TOF) is the most common cyanotic congenital heart defect, typically requiring early treatment in infancy. Untreated TOF is associated with poor survival, with most uncorrected patients not surviving beyond the third decade. Here, we present a unique case of an 86-year-old female with uncorrected TOF who underwent a transcatheter aortic valve replacement (TAVR) procedure due to severe aortic stenosis (AS). The patient's TOF was identified during infancy, and she was categorized as an acyanotic "pink baby." Notably, the first palliative surgery for TOF was performed in 1944, when the patient was seven years old. The patient never underwent corrective surgery for TOF and continued to lead a symptom-free life until she developed severe AS later in life. The TAVR procedure significantly improved her symptoms, illustrating the importance of considering alternative etiologies for symptoms in elderly patients with uncorrected TOF and AS. In addition, we underscore the anesthetic management during TAVR, specifically highlighting the challenges addressed, such as the utilization of general anesthesia with transesophageal echocardiography (TEE) and the placement of a pulmonary artery (PA) catheter.

## Introduction

Tetralogy of Fallot (TOF) is the most common cyanotic congenital heart defect, characterized by a combination of ventricular septal defect (VSD), right ventricular outflow obstruction, right ventricular hypertrophy, and an overriding aorta. Early diagnosis and intervention are essential for improving outcomes, as most patients require treatment in infancy and exhibit symptoms at an early age. Untreated TOF is associated with dismal survival rates, with the majority of uncorrected patients failing to survive beyond their third decade [1].

Within this context, we present an extraordinary case of an 86-year-old female with unrepaired TOF who underwent a transcatheter aortic valve replacement (TAVR) procedure due to severe aortic stenosis (AS). This review highlights the intricate challenges and considerations in anesthetic management faced by the medical team during the successful TAVR procedure in an elderly patient with long-standing uncorrected TOF.

## Case presentation

An 86-year-old female with uncorrected TOF presented with chest pain, worsening shortness of breath, and New York Heart Association (NYHA) functional class II-IV symptoms. Surgical repair was never pursued due to her mild TOF and acyanotic "pink baby" status. On admission, a 2D echocardiogram revealed an ejection fraction (EF) of 70%, right ventricular systolic pressure (RVSP) of 59, a moderately dilated left atrium, a small VSD, severe AS with a peak velocity of 4.9 m/s, an aortic valve area (AVA) of 0.5 cm², and a mean gradient of 41 mmHg across the AV. Cardiac catheterization showed normal coronaries, with RVSP measured at 83/13, pulmonary artery (PA) at 58/26, and a gradient of 25 mmHg across the pulmonic valve indicating mild pulmonic stenosis.

Given her age, complex anatomy, frailty, and a 6.4% predicted risk of mortality with surgical aortic valve replacement (AVR) according to the Society of Thoracic Surgeons (STS) score, the patient opted against surgery involving cardiopulmonary bypass (CPB). She accepted the potential risks, including the possibility of a hemorrhage that could be addressed with a sternotomy. Despite the limited interventions available in case TAVR failed, the patient chose to proceed with the procedure to alleviate her symptoms.

With the patient's preference to avoid CPB but acceptance of sternotomy in the case of a hemorrhage, a TAVR procedure was planned under general anesthesia with transesophageal echocardiography (TEE) guidance to address the severe AS suspected as the cause of her recent symptoms. A PA catheter was placed from the groin. Pressures obtained showed a gradient across a pulmonic valve of 25 mmHg, and oxygen saturations were obtained, showing a shunt fraction (Qp:Qs) of 1.7 with a VSD.

**Figure 1:**
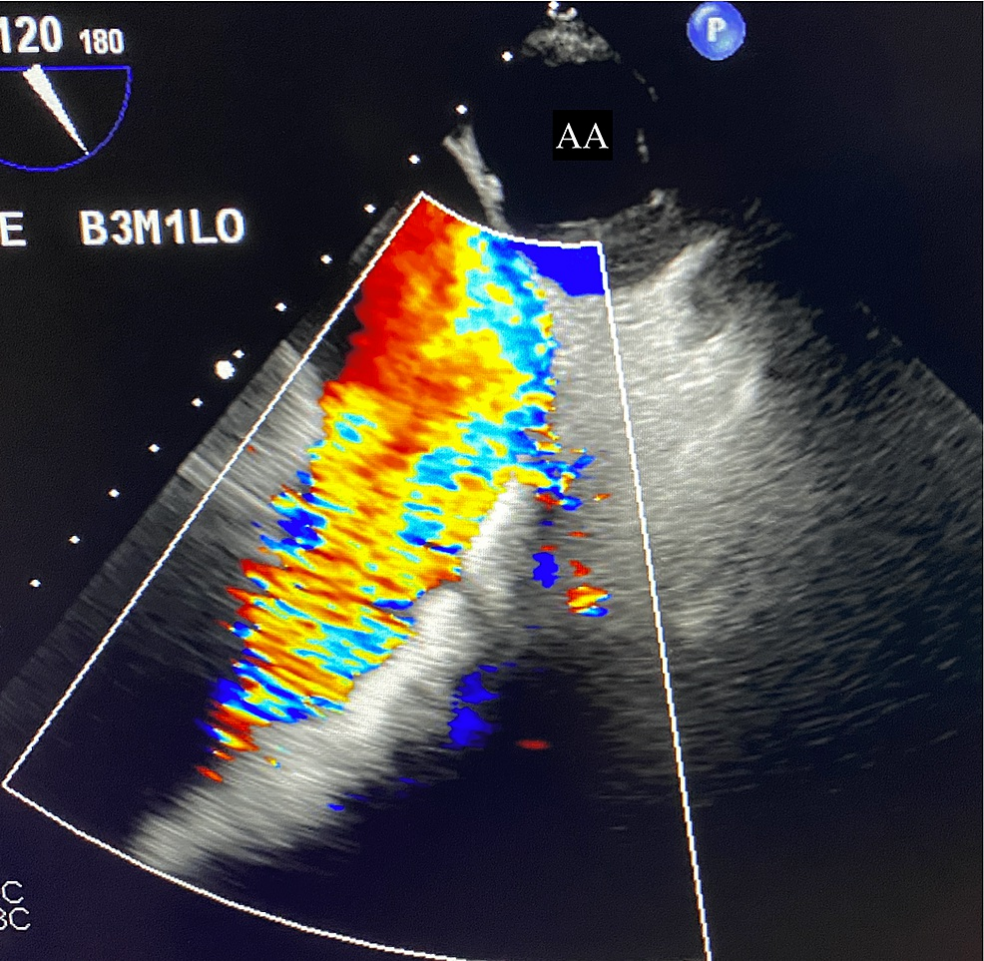
Pre-transcatheter aortic valve replacement (TAVR). Upper esophageal view of the pulmonic valve showing subpulmonic stenosis

**Figure 2:**
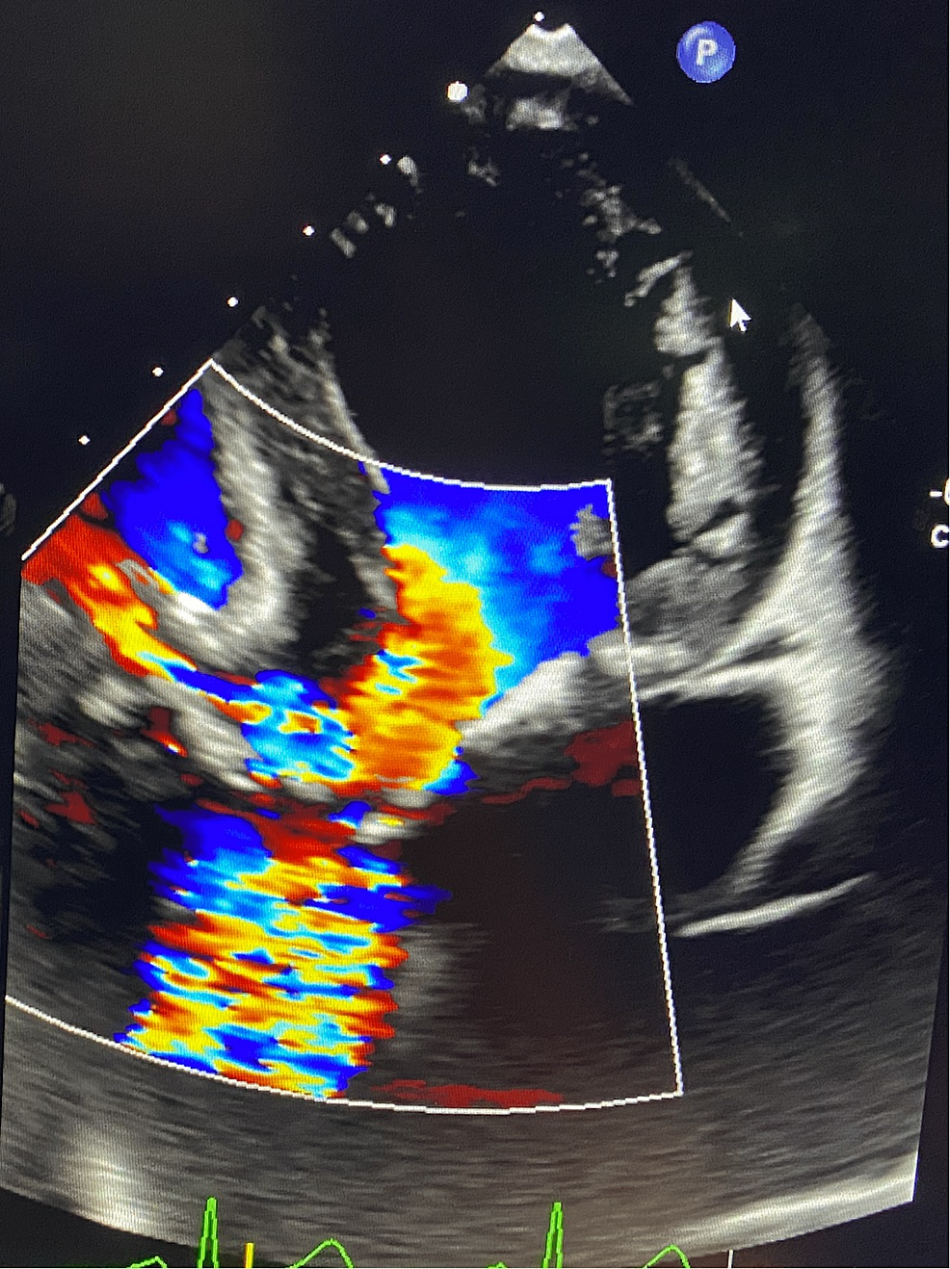
Pre-transcatheter aortic valve replacement (TAVR). Turbulent flow through the aortic valve signifying aortic stenosis with a left-to-right shunt through the VSD

**Figure 3:**
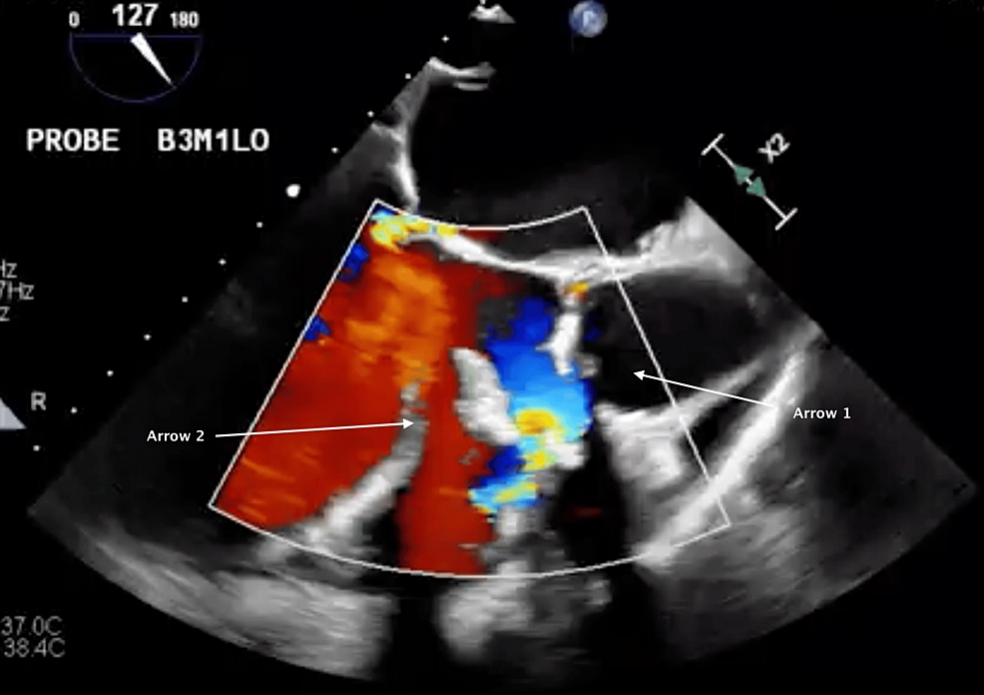
Arrow 1 highlights the aorta positioned atop the ventricular septal defect, as indicated by Arrow 2, showcasing the overriding aorta in tetralogy of Fallot (TOF)

**Figure 4:**
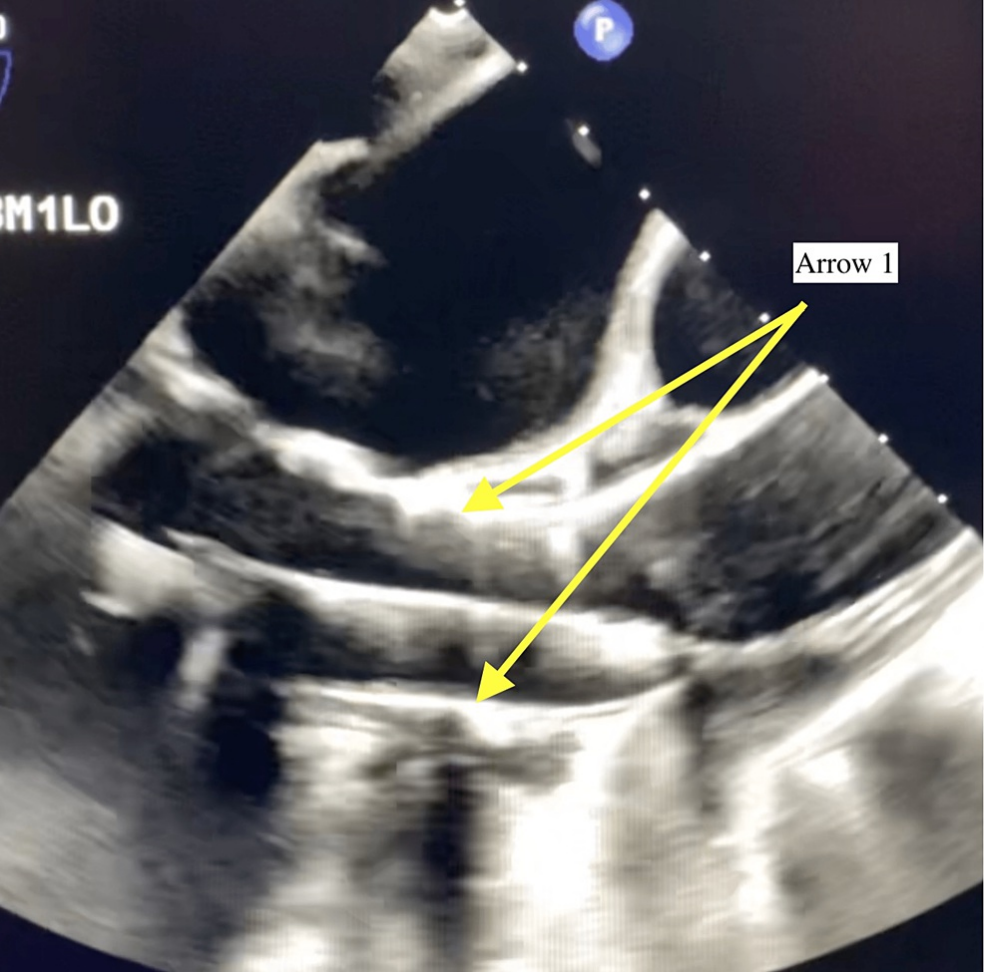
Deployment of the 26-mm Sapien S3 valve shown by Arrow 1

**Figure 5:**
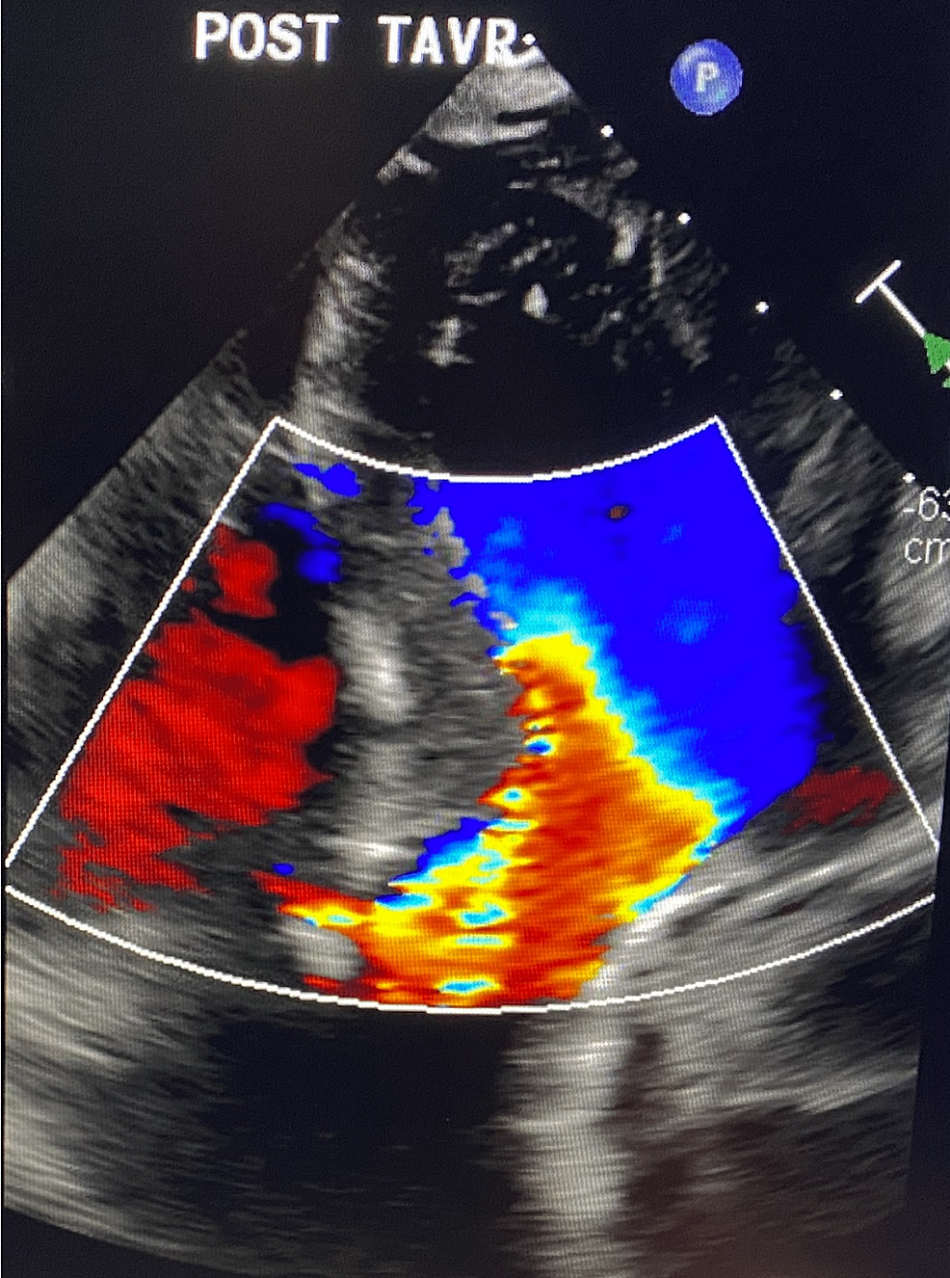
Post-TAVR: Laminar flow through aortic valve signifying the resolution of aortic stenosis. Small residual VSD with a left-to-right shunt

Intraoperative TEE revealed subpulmonic stenosis (Figure 1) and turbulent flow across the AV, indicating AS with a left-to-right shunt through the VSD (Figure 2). In the long-axis view, Figure 3 reveals the overriding aorta positioned atop the VSD. Video 1 provides a detailed visual of the mid-esophageal four-chamber view, illustrating the dynamic flow through the VSD and showcasing the overriding aorta. After deploying the 26-mm Sapien S3 valve via right transfemoral access (Figure 4), the laminar flow through the AV indicated a successful resolution of the AS, with a small residual VSD and left-to-right shunting observed (Figure 5).

**Video 1:** Echocardiogram: mid-esophageal four-chamber view capturing the dynamic flow through the ventricular septal defect (VSD) and showcasing the overriding aorta

The post-TAVR echocardiogram showed an EF of 60%, a prosthetic AVA of 1.7 cm², a peak velocity of 2.6 m/s, an AV mean gradient of 9 mmHg, and a dimensionless index of 0.6. Notably, there was no evidence of a paravalvular or central leak. Aortic root angiography confirms the patency of coronary vessels, the absence of significant aortic regurgitation, and an excellently placed valve. The valve and delivery apparatus were then removed, and a thorough interrogation with TEE was performed. The outcome was satisfactory, so the delivery sheath was removed, and protamine was administered. Pulmonary pressures remained stable, allowing for the removal of the Swan-Ganz catheter. The patient developed a left bundle branch block, prompting the securement of a temporary pacemaker. The patient tolerated the procedure well under general anesthesia and was subsequently extubated in the ICU.

The patient's symptoms markedly improved, and she was discharged with scheduled outpatient follow-up. This successful TAVR procedure provided valuable insights into the management of severe AS in elderly patients with uncorrected TOF, offering a potential alternative to surgical repair and enhancing the patient's overall clinical condition.

## Discussion

TOF typically presents in infancy as a cyanotic congenital heart defect, with rare cases presenting after middle age [1]. The primary causes of mortality in TOF patients are hypoxic spells and cerebrovascular accidents during early life, while brain abscesses become more prevalent in older patients [2]. Only a small percentage of patients, about 3%, survive beyond the age of 40 years [2]. The oldest recorded survivor of unrepaired TOF is an 87-year-old woman [3]. Despite such cases, there is no documented instance of an unrepaired TOF patient undergoing TAVR.

Notably, only a minority of TOF patients, estimated at around 5%, do not exhibit central cyanosis, as seen in our patient [4]. The severity of the obstruction in the right ventricular outflow tract (RVOT) plays a significant role in the pathophysiology, with systemic vascular resistance (SVR) influencing the direction of shunting from right to left [5]. Early diagnosis often involves detecting cyanosis resulting from right-to-left shunting due to increased resistance in the RVOT compared to the aorta [2]. "Pink TOF" occurs when left-to-right shunting across the VSD leads to the absence of peripheral cyanosis [2].

In this case, there was a concern about the potential shift from a left-to-right shunt to a right-to-left shunt through the VSD. Patients with AS typically exhibit hypertrophied left ventricles, leading to elevated peak and mean pressure gradients across the AV [6]. Given the concomitant right ventricular hypertrophy associated with TOF, resulting in the characteristic "boot-shaped heart" appearance, it was crucial to ensure that relieving the AV obstruction did not generate higher pressures in the right ventricle than the left, potentially causing a right-to-left shunt [7]. As a result, the TAVR procedure was carried out under general anesthesia with TEE and PA catheter placement to enable real-time assessment of biventricular function and to manage pulmonary vascular resistance. Although rare cases of untreated TOF in the elderly have been reported, no specific management considerations for TAVR in these patients have been documented.

The primary anesthetic goal in this case focused on preventing right-to-left shunting across the VSD. General endotracheal anesthesia (GETA) was selected despite concerns about potential hemodynamic fluctuations associated with general anesthesia to maintain airway control, monitor cardiac function, and prepare for potential sternotomy in case of hemorrhage during the procedure. The patient opted not to undergo CPB.

At the outset of the procedure, the patient had two large bore IVs and an arterial line in place. A cardiac-stable induction was successfully achieved using fentanyl (25 mcg) and etomidate (16 mg). Subsequently, the cardiac surgeon introduced a PA catheter through the groin to monitor PA pressures. For maintenance, sevoflurane and atracurium (30 mg) were administered, with careful ventilation management to prevent excessive positive airway pressure and minimize any reduction in the pulmonary blood flow that might lead to increased pulmonary vascular resistance. A continuous infusion of phenylephrine was administered to maintain SVR prior to valve placement. Adequate intravascular volume was maintained by preloading the patient with 500 ml of lactated Ringer's solution, which further contributed to the patient's hemodynamic stability.

## Conclusions

This complex case represents one of the oldest known living patients with unrepaired TOF undergoing TAVR as reported in the literature. The feasibility of employing alternative anesthesia techniques in this case proved pivotal in ensuring a successful outcome. The decision to opt for GETA with TEE and PA catheter, despite potential hemodynamic considerations, was crucial for maintaining airway control and facilitating comprehensive cardiac monitoring. This choice was particularly significant given the patient's preference to avoid CPB but acceptance of sternotomy in the case of major bleeding. This successful intervention not only provided significant relief of symptoms but also contributes valuable insights into the management of severe AS in elderly patients with uncorrected TOF.
